# The Dual Role of TGF-β and Hypoxia on MMP14-Mediated Invasion in PC3 Cells

**DOI:** 10.1007/s12013-026-02102-3

**Published:** 2026-06-25

**Authors:** Derya Okuyan, Hanife Aktaş, Fatma Poyrazlı, Sümeyye Aydoğan Türkoğlu

**Affiliations:** 1 Department of Veterinary Medicine, Susurluk Agriculture & Forestry Vocational School, Balıkesir University, Susurluk, Türkiye; 2 Department of Molecular Biology and Genetics, Institute of Science, Balıkesir University, Balıkesir, Türkiye; 3 Department of Molecular Biology and Genetics, Faculty of Science and Literature, Balıkesir University, Balıkesir, Türkiye

**Keywords:** MMP14, Hypoxia, TGF-β, Promoter regulation, PC3 cells, COL1A1, Metastasis

## Abstract

The MMP14 gene, which encodes the only membrane-bound collagenase, is highly expressed in malignant tumors and plays a decisive role in invasiveness. This study investigated the transcriptional regulation of MMP14 by the TGF-β cytokine and hypoxia in PC3 prostate carcinoma cells. Bioinformatic analysis confirmed the presence of key regulatory elements in the MMP14 promoter, specifically the HRE (for HIF-1α) and SMAD binding sites. Three 5′ deletion fragments of the MMP14 promoter (P1: −1251/+75; P2: −649/+75; P3: −176/+75) were cloned for functional analysis. PC3 cells were treated with TGF-β, the hypoxia-mimetic CoCl_2_, and the combination. Gene and protein expression were analyzed using Real-Time PCR and Immunofluorescence Cytochemistry (IFC), respectively, while promoter activities were assessed via a Luciferase reporter assay. COL1A expression was also evaluated to explore its association with MMP14-mediated extracellular matrix remodeling. Hypoxia emerged as the dominant inductive factor. MMP14 mRNA and protein levels were significantly elevated under hypoxic and combined TGF-β/CoCl_2_ conditions. Promoter analysis revealed that the full-length P1 construct was consistently upregulated by all treatments. However, the shorter P2 and P3 fragments exhibited a decrease in activity with TGF-β alone, suggesting a complex, region-specific regulation predominantly influenced by hypoxic signaling. Critically, COL1A1 mRNA expression was observed in hypoxic groups with elevated MMP14 levels, indicating a possible inverse relationship between MMP14 induction and COL1A-associated extracellular matrix remodeling. Hypoxia is the primary driver of MMP14 induction and protein accumulation in PC3 cells, with TGF-β acting as a complex co-regulator whose activating effect is potentiated under low-oxygen stress. These findings highlight the contribution of the HIF/MMP14 axis to invasion-associated molecular regulation in prostate cancer.

**Supplementary Information:**

The online version contains supplementary material available at https://doi.org/10.1007/s12013-026-02102-3.

## Introduction

The extracellular matrix (ECM) is a complex structure formed by tissue cells in the body and components secreted from these cells [1, 2]. The main components of the ECM are collagens, laminins, glycoproteins, elastin, and fibronectin. Furthermore, fibril-forming collagen type I and cartilage collagen type II are the main components of the ECM in most tissues [1]. The matrix metalloproteinase (MMP) family is an important group of calcium (Ca^+ 2^) and zinc (Zn^+ 2^)-dependent neutral endopeptidases that have the ability to degrade ECM and basement membrane components [3–5]. MMP14 (MT1-MMP), one of the membrane-type members of the MMP family, which has 24 members in humans, is localized to the 14q11.2 region of chromosome 14 [4, 6]. The MMP14 gene is highly expressed in tissues such as the urinary bladder, placenta, gallbladder, and endometrium, while its expression is normal in tissues such as the prostate, skin, lung, brain, breast, ovary, and testis. MMP14, a proteinase, is regulated by transcriptional and posttranscriptional modifications. Epithelial-mesenchymal transition (EMT), a crucial stage in cancer metastasis and embryonic development, is frequently utilized by malignant cells for growth and metastasis induction [7]. MMPs promote tumor development by regulating invasiveness and the growth of metastatic tumors, and MMP14, a membrane-associated collagenase, plays a decisive role in these processes [8]. However, cancer cells that gain mobility encounter obstacles to cell migration from fibrillar collagens, particularly type I collagen, which are components of the ECM. MMP14 contributes to degradation/remodeling of fibrillar collagen barriers associated with migration. Therefore, MMP14 increases cell migration in cancer cells [6, 9].

Transforming growth factor (TGF)-β is a key regulator of EMT and a key cytokine that facilitates cancer progression through changes in the tumor and its microenvironment. High TGF-β expression has been associated with poor prognosis in cancer patients. Genetic and epigenetic changes in cancer cells alter TGF-β function, leading to the emergence of aggressive and metastatic properties [10].

Hypoxia, a condition caused by decreased oxygen levels, paves the way for numerous changes in cells [11]. Cells adapt to hypoxic conditions by inactivating or activating specific genes. This allows tissues and cells to survive under varying environmental factors. The HIF-1α gene is activated under hypoxic conditions and interacts with enzymes and other transcription factors to control vascularization and tissue growth. HIF-1α, a transcription factor, binds to the HRE (5’-RCGTG-3’) sequence located in the promoter region of relevant genes and plays a role in gene expression by responding to hypoxia. Hypoxia increases proliferation, angiogenesis, and metastasis signaling in tumor cells. Furthermore, there is clinical evidence that tumor diameter increases with increasing hypoxia [12]. HIF also plays a role in regulating the adaptation of cancer cells to EMT [13].

Transforming Growth Factor-β (TGF-β) and hypoxia are two major components of the tumor microenvironment that cooperatively regulate invasion, extracellular matrix (ECM) remodeling, and metastatic progression. TGF-β signaling promotes epithelial–mesenchymal transition (EMT), fibrosis, and matrix deposition through SMAD-dependent pathways, whereas hypoxia activates Hypoxia-Inducible Factor-1α (HIF-1α)-mediated transcriptional responses associated with tumor progression and invasiveness [10–13]. Since both pathways influence extracellular matrix remodeling and metastasis-associated genes, their combined contribution to MMP14 regulation is of particular interest. In this context, COL1A1 (Collagen Type I Alpha 1), a major component of fibrillar type I collagen within the ECM, was also evaluated. COL1A1 plays an important role in matrix organization and tumor-associated stromal remodeling, and its expression may reflect changes associated with MMP14-mediated extracellular matrix dynamics [14].

This study investigates the regulatory role of the cytokine TGF-β on the expression of the MMP14 gene, a factor frequently implicated in various cancer studies. Although MMP14 expression is typically low in non-malignant cells, it is significantly elevated in cancerous tissues. Given that MMP14 is the sole membrane-bound collagenase, it is hypothesized to play a critical and decisive role in enhancing tumor invasiveness and promoting the progression of metastatic disease. Importantly, the tumor microenvironment is often characterized by hypoxia, a condition known to be a potent inducer of genes associated with invasion and metastasis, including certain Matrix Metalloproteinases. Therefore, this research specifically aimed to elucidate the regulatory mechanism governing MMP14 expression under these critical conditions. The investigation assessed the impact of TGF-β on MMP14 gene expression under both normoxic conditions and conditions of chemically mimetic hypoxia induced by CoCl_2_.

## Material Methods

### Culturing of Cells and MTT Assay

In this study, PC3 human prostate cancer cells, originally established from a bone metastasis of stage IV prostate adenocarcinoma, were employed. The cell line was kindly supplied by Prof. Dr. Kemal Sami Korkmaz from the Department of Bioengineering, Ege University, Turkey. Cells were maintained in DMEM/F-12 growth medium (EuroClone, Italy) supplemented with 10% heat-inactivated fetal calf serum (FCS; Gibco, UK). Cultures were incubated at 37 °C in a humidified atmosphere containing 5% CO₂ and propagated in 75 cm² culture flasks (Sarstedt, Germany). Subculturing was routinely carried out two to three times per week to maintain optimal growth conditions. To determine non-toxic working concentrations for subsequent experiments, an MTT cell viability assay was conducted using two agents: the cytokine TGF-β1 (Cell Signaling Technology, USA) and the chemical hypoxia mimic cobalt chloride (CoCl₂). The assay results were used to establish safe concentration ranges for further cellular treatments.

Cells were distributed in 96-well plates with 1 × 10⁴ PC3 cells per well, and the plates were labeled for different time intervals. After 24 h, culture medium was replaced with serum-reduced medium containing 0.1% BSA. One hour after the medium change of the cells that would receive TGF-β and CoCl_2_, CoCl_2_ was applied to a final volume of 150 µM to create chemically induced hypoxia groups. To establish the TGF-β treatment group, cells were treated with TGF-β1 at a final concentration of 20 ng/mL [15, 16].

For the combined treatment group under chemical hypoxic conditions, cells were treated with TGF-β1 (20 ng/mL) together with CoCl₂ at a final concentration of 150 µM. Studies in hepatocellular, prostate, and colorectal cancers demonstrated that the CoCl₂-mediated hypoxia model consistently triggers reproducible hypoxia-associated molecular responses [17–21]. Cells were incubated for 1, 3, 6, 24, and 48 h under the respective treatment conditions. At the end of each incubation period, MTT solution was added to each well to a final concentration of 0.5 mg/mL, followed by incubation for 4 h at 37 °C in a humidified atmosphere containing 5% CO₂. At the end of the process, the medium was removed and isopropanol (containing 0.004 M HCl) was applied. Then, absorbances were measured at 550 nm wavelength [22].

### Analysis of MMP14 Promoter Regulation

To explore the regulatory mechanisms governing MMP14 gene expression, three sequential 5′ deletion variants of the human MMP14 promoter were produced through a PCR-based amplification strategy (adapted from [15]). The resulting truncated fragments—designated P1 (− 1251/+75), P2 (− 649/+75), and P3 (− 176/+75)—represented progressive deletions upstream of the transcription initiation site (TSS). Each promoter fragment was amplified using specific primer pairs and initially inserted into the pGEM-T Easy vector (Promega, LIE) via T/A cloning. Verified recombinant plasmids were subsequently subcloned into the pMetLuc reporter construct. The sequence integrity of all constructs was confirmed by DNA sequencing.

Transient transfection experiments were carried out using the calcium phosphate precipitation approach to assess the transcriptional potential of each promoter construct. To normalize transfection efficiency and establish baseline activity, 0.5 µg of the pMetLuc control plasmid was included as a positive reference control, whereas an empty pMetLuc reporter vector (0.5 µg) was used as a negative control. Following transfection, cells were exposed to TGF-β1 (20 ng/mL) and cobalt chloride (CoCl₂, 150 µM), both individually and in combination, to simulate cytokine signaling and hypoxic conditions. After 48 and 72 h of incubation, culture supernatants were harvested, and secreted luciferase (MetLuc) and alkaline phosphatase (SEAP) activities were quantified using the Ready-To-Glow™ Secreted Luciferase assay kit (Takara, China), with SEAP activity serving as an internal control for normalization of transfection efficiency [15–18, 21]. Luminescence was detected with a luminometer (Thermo, USA). All assays were conducted in triplicate and independently reproduced three times to ensure experimental reliability.

### Quantitative RT-PCR (qRT PCR)

For quantitative gene expression profiling, PC3 cells were plated at a density of 2 × 10⁶ cells per 25 cm² culture flask (Sarstedt, Germany). After seeding, cells were exposed to TGF-β1 (20 ng/mL), CoCl₂ (150 µM), or their combination and incubated for 1, 3, 6, or 24 h. Following treatment, total RNA was extracted from the harvested cell pellets using the GeneJET RNA Purification Kit (Thermo Scientific, USA) according to the manufacturer’s protocol. Quantitative real-time PCR analysis was performed using a Roche LightCycler^®^ 480 Real-Time PCR System (Roche Diagnostics, Switzerland) to determine MMP14 and COL1A1 transcript levels under different treatment conditions and time intervals. Primer sequences used in the amplification reactions are listed in Supplementary Table 1. Each reaction was conducted in a 96-well plate with a total volume of 12.5 µL, containing 1 µL of cDNA template, 6.25 µL of SYBR Green PCR Master Mix (Ampliqon, Denmark), 0.5 µL of both forward and reverse primers (50 ng/µL each), and 4.25 µL of nuclease-free water [22]. Relative mRNA expression levels were quantified using the 2^⁻ΔΔCt^ (Livak) method [23]. Statistical analyses of gene expression data were carried out using the Minitab 14 software package.

### Immunofluorescence Staining

PC3 cells were seeded into 12-well plates (SPL Life Sciences, Korea) at a density of 1.25 × 10⁵ cells per well. After cell attachment, cultures were exposed to TGF-β1 (20 ng/mL) and cobalt chloride (CoCl₂, 150 µM) for 24 h. Following incubation, the treatment media were discarded, and cells were rinsed briefly with phosphate-buffered saline (PBS; Sigma, USA/Canada). Fixation was then performed using 4% paraformaldehyde for 15 min at room temperature. After fixation, the paraformaldehyde solution was removed, and cells were washed once again with PBS [16, 19, 20]. Since MMP14 (MT1-MMP; Cat. No. sc-377097, Santa Cruz Biotechnology, Dallas, TX, USA) is a membrane-associated protein, permeabilization with Triton X-100 was omitted. To block nonspecific antibody binding, 10% bovine serum albumin (BSA) prepared in PBS was added to each well and incubated for 30 min. After a brief wash, the cells designated for MMP14 analysis were incubated with the primary antibody (diluted 1:50 in 1% BSA/PBS) for 1 h. Following another wash step, cells were treated with Alexa Fluor 488-conjugated secondary antibody (Thermo Fisher, USA), diluted 1:100 in 1% BSA, and incubated for 1 h at 25 °C. Nuclei were subsequently counterstained with 4′,6-diamidino-2-phenylindole (DAPI). Fluorescent signals were then visualized and captured using an Olympus DP72/ED200 fluorescence microscope (Japan).

### Statistical Analysis

Differences among experimental groups were evaluated using one-way analysis of variance (ANOVA) performed with Minitab version 15. Results were considered statistically significant when the p-value was ≤ 0.05.

## Results

### In Silico Analysis of MMP14 Promoter

**Fig. 1:**
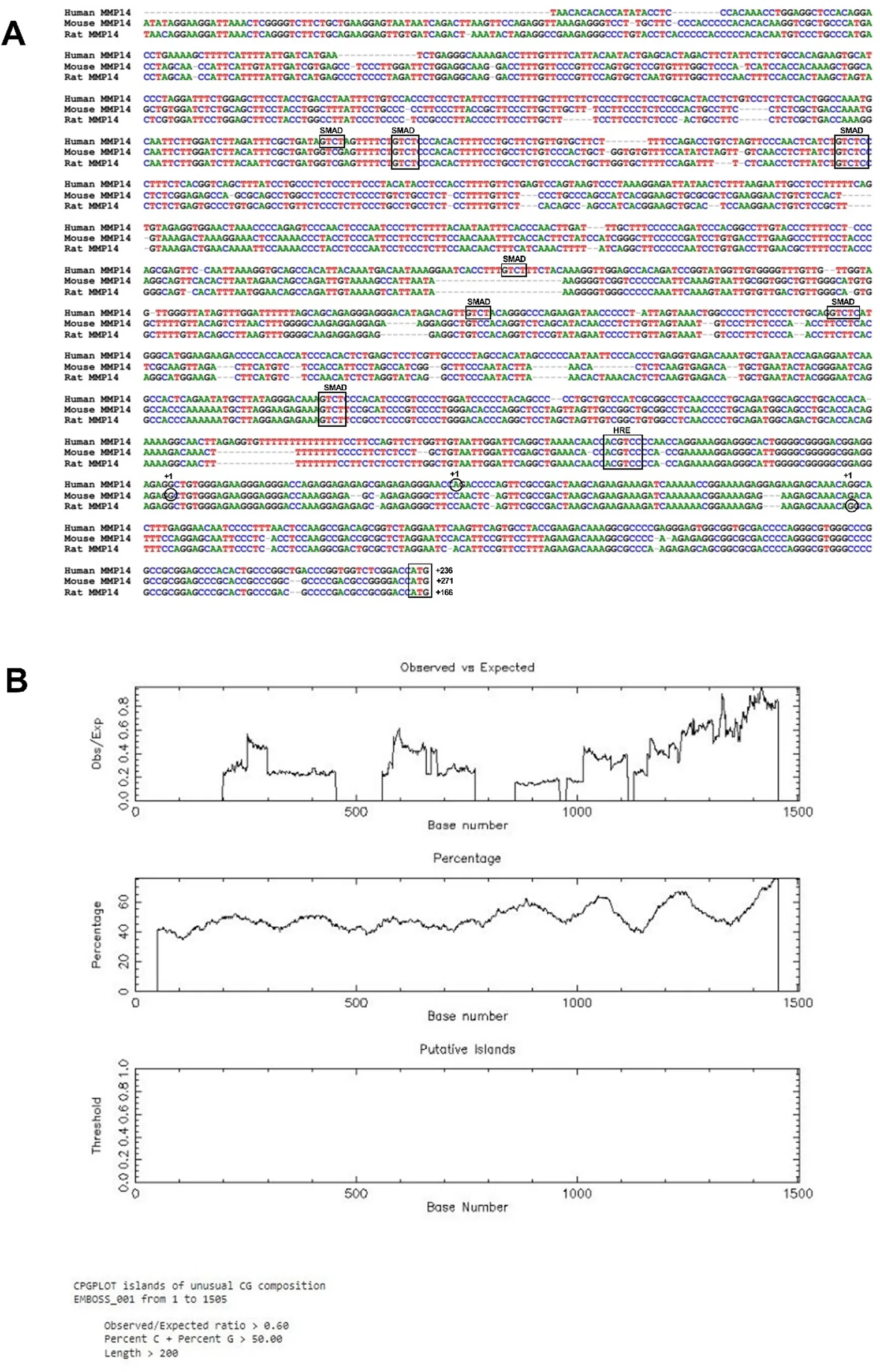
In silico analysis of MMP14 promoter. A: Comparison of human, mouse and rat MMP14 promoter regions. The + 1 base on the Human, Mouse and Rat promoters is marked with the “Ο” symbol. The HRE and SMAD regions on the human promoter are also marked. Additionally, the HRE and SMAD regions common to the mouse and rat promoter are also marked on the sequences. B: GC islands belonging to the MMP14 promoter region

The MMP14 promoter sequences from three distinct mammalian species human (NC_000014.9), mouse (NC_000080.7), and rat (NC_051350.1) were subjected to analysis and subsequent alignment utilizing the BioEdit software. The region of interest was defined as 1500 base pairs extending in the 5’-direction from the ATG translational start codon. Specifically, the following regions were analyzed: the − 1236 / +236 region for the human MMP14 gene, the − 1229 / +271 region for the mouse MMP14 gene, and the − 1334 / +166 region for the rat MMP14 gene. The transcription start site (+ 1 base) on the human, mouse, and rat promoters is marked with the “O” symbol. A comparative analysis revealed significant conservation of regulatory elements. Specifically, the Hypoxia Response Element (HRE), situated at positions − 95/-89 in the human promoter, was also conserved and identified in the mouse (-47/-41) and rat (-154/-148) promoters. Furthermore, a set of SMAD (Sma and Mad-related) binding sites located at -259/-255, -885/-881, and − 951/-947 in the human MMP14 promoter demonstrated positional conservation in the mouse (-198/-194; -770/-766; -841/-837) and rat (-308/-304; -879/-875, -948/-940) promoters. In addition to these conserved sites, several other SMAD binding sites were identified. In the human MMP14 promoter, these sites are found at -963/-959, -905/-901, -585/-581, -474/ -470, and − 451/-447. Correspondingly, the mouse MMP14 promoter contains SMAD sites at -1206/-1202, -725/-721, -663/-659, -424/-420, and − 380/-376. Lastly, the rat MMP14 promoter features SMAD binding sites at -1311/-1307, -774/-770, -497/-493, and − 424/-420 (Fig. 1A). This comparative analysis revealed that the MMP14 promoter regions exhibited a high degree of conservation. Specifically, the promoter regions between human and rat showed 82% sequence similarity, while the similarity between human and mouse was slightly lower at 80%. The highest level of identity was found between the rat and mouse MMP14 promoter regions, which shared 88% similarity. These findings underscore the evolutionary conservation of the MMP14 transcriptional regulatory elements across these species. To predict the presence of Guanine and Cytosine (GC) islands within the potential MMP14 promoter region, an in silico analysis was performed using the EMBL-EBI (European Molecular Biology Laboratory – European Bioinformatics Institute) CPGPlot tool (available at https://www.ebi.ac.uk/Tools/seqstats/emboss](https://www.ebi.ac.uk/Tools/seqstats/emboss_cpgplot/*).* The analysis determined that the GC content of the region was approximately 50% (Fig. 1B). Critically, the standard threshold for defining a true GC island a feature often associated with active housekeeping gene promoters typically requires a GC ratio of 70% to 80% or higher. Therefore, the observed 50% content suggests the absence of a defined GC island in the analyzed MMP14 promoter segment.

### Transcriptional Regulation Analysis of the MMP14 Promoter

**Fig. 2:**
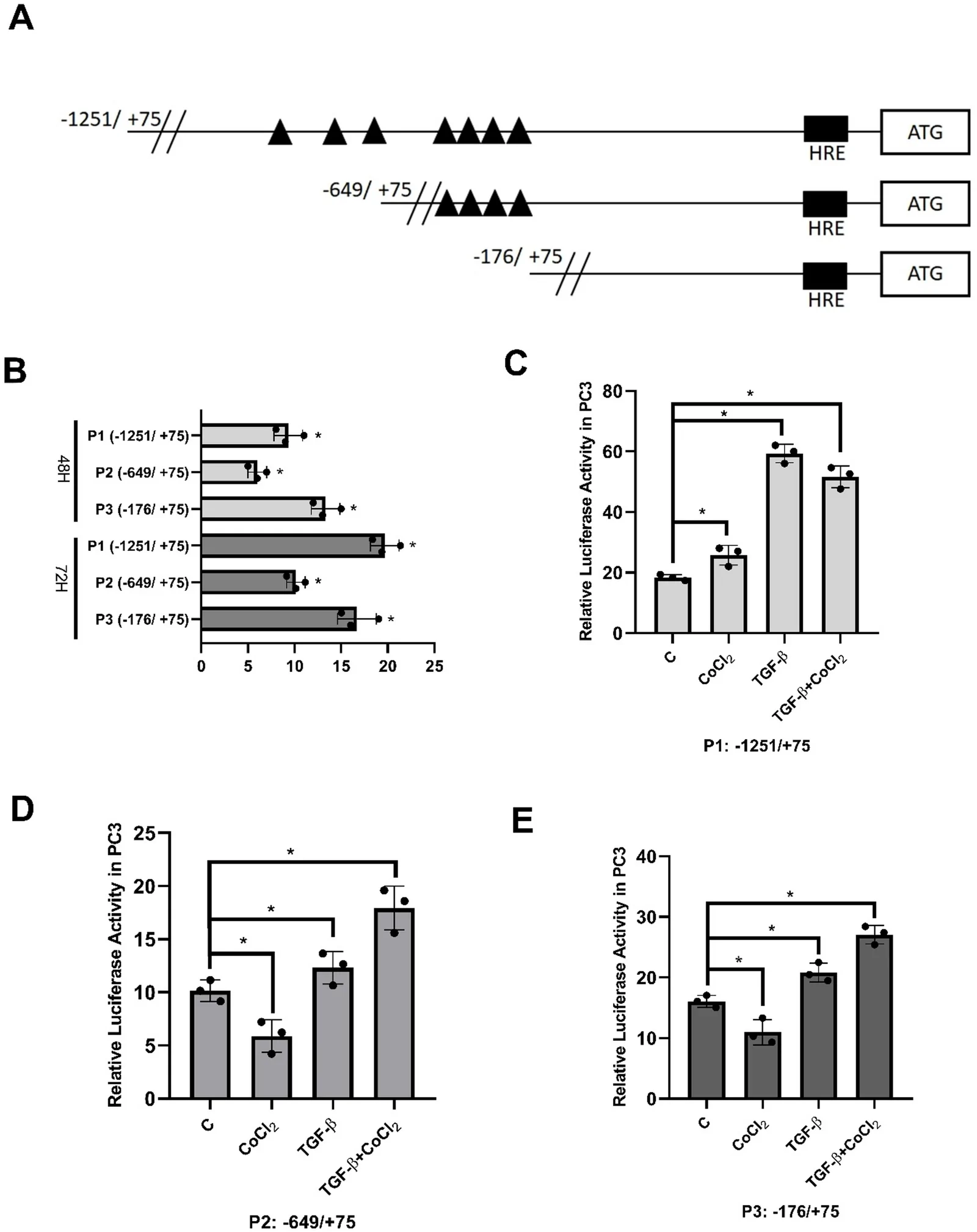
Determination of Transcriptional Regulation of the MMP14 Promoter. A: Graphical representation of the SMAD and HRE regions on the P1 (− 1251/+75), P2 (− 649/+75), and P3 (− 176/+75) promoter fragments. HRE regions on the sequences are marked with the symbol █, and SMDA regions are marked with ▲. B: Basal activities of the P1 (− 1251/+75), P2 (− 649/+75), and P3 (− 176/+75) promoter fragments at 48 and 72 h. C: Effects of TGF-β1 (20 ng/mL) and chemical hypoxia (CoCl₂) on the transcriptional activity of the P1 (− 1251/+75) promoter fragment in PC3 cells D: Effects of TGF-β1 (20 ng/mL) and chemical hypoxia (CoCl₂) on the transcriptional activity of the P2 (− 649/+75) promoter fragment in PC3 cells. E: Effects of TGF-β1 (20 ng/mL) and chemical hypoxia (CoCl₂) on the transcriptional activity of the P3 (− 176/+75) promoter fragment in PC3 cells. Data are presented as mean ± SD. (*p ≤ 0.05)

To precisely characterize the transcriptional regulation of the MMP14 promoter, a series of three different 5′ deletion mutants were generated: P1 (− 1251/+75), P2 (− 649/+75), and P3 (− 176/+75). Bioinformatic analysis of the full-length promoter region (− 1251/+75) was conducted to identify key regulatory elements. Specifically, the analysis involved mapping the locations of key regulatory elements within the promoter region: the Hypoxic Response Element (HRE), which mediates the cellular response to hypoxia, and the SMAD transcription factor binding sites, which govern the SMAD-dependent transcriptional response to TGF-β cytokine signaling. HRE sites situated on the P1 (− 1251/+75), P2 (− 649/+75), and P3 (− 176/+75) promoter fragments are schematically indicated with a black square (█), while SMAD binding sites are designated by a black triangle (▲, Fig. 2A). Following this mapping, the basal transcriptional activity of each constructed promoter fragment was quantified in PC3 prostate carcinoma cells. The P1 fragment exhibited the highest basal activity, suggesting that the regulatory elements within the − 1251 to − 649 region are crucial for baseline MMP14 expression. Conversely, the P2 (− 649/+75) fragment exhibited the lowest transcriptional activity among all constructs (Fig. 2B). The effects of TGF-β (500 U/mL [20 ng/mL]) on these promoter fragments were examined under both normoxic and hypoxic conditions. The activity of the P1 and P2 promoters was found to be increased following exposure to both TGF-β alone and the combined TGF-β and hypoxic conditions (Fig. 2C and D). However, the shortest promoter fragment, P3, which is hypothesized to lack critical SMAD binding sites, showed a decrease in activity specifically under hypoxia (Fig. 2E). These results indicate a differential, region-specific response to TGF-β and hypoxia, highlighting the importance of the upstream regulatory sequences (present in P1 and P2) for maximal response.

### Validation of Hypoxia and MMP14 Gene Regulation Analysis in PC3 Cells

**Fig. 3:**
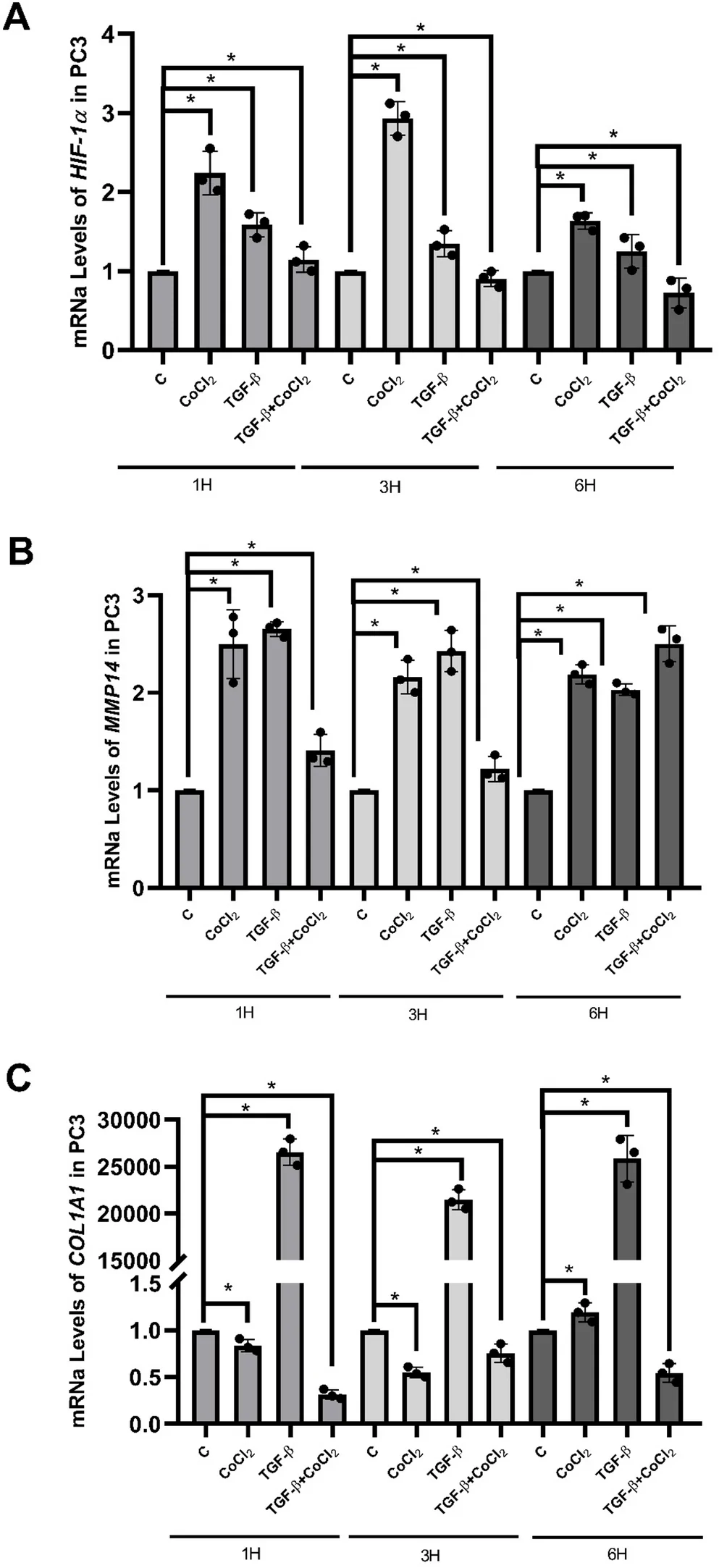
Regulation of HIF-1α, MMP14, and COL1A1 Gene Expression in PC3 Cells by Hypoxia and TGF-β Signaling. The mRNA expression levels of target genes were quantified in PC3 prostate cancer cells following treatment for 1, 3, and 6 h. Cells were exposed to four distinct conditions: Normoxia (control), Chemically Mimetic Hypoxia (CoCl_2_), TGF-β treatment (20 ng/ml), and the combination of CoCl₂-induced chemical hypoxia and TGF-β. Relative mRNA levels were determined by quantitative Real-Time PCR (qPCR) and normalized to an internal control gene human β2-microglobulin (hβ2). **A**: HIF-1α Gene Expression. Demonstrates the expected significant upregulation of HIF-1α transcripts under CoCl₂-induced chemical hypoxia (both CoCl_2_ alone and combined with TGF-β), confirming the success of the hypoxic induction model. **B**: MMP14 Gene Expression. Shows the relative changes in MMP14 mRNA levels across all treatment groups. **C**: COL1A1 Gene Expression. Illustrates the transcriptional regulation of the COL1A1 gene (Collagen Type 1 Alpha) under the influence of hypoxia and TGF-β. (* *p* ≤ 0.05)

To validate the chemically induced hypoxic model in the PC3 cell line, we first confirmed the upregulation of the master transcription factor, Hypoxia-Inducible Factor-1α (HIF-1α), following treatment with the hypoxia-mimetic agent CoCl₂. Following the isolation of total RNA from both normoxic control and CoCl₂-treated hypoxia-mimetic cell cultures, HIF-1α transcripts were analyzed by quantitative Real-Time PCR utilizing specific primers. Comparison of HIF-1α mRNA levels revealed a significant upregulation in expression at the 1, 3, and 6-hour time points (Fig. 3A), successfully confirming the induction of the hypoxic state.

The regulatory effects of hypoxia and TGF-β on the mRNA expression of MMP14, and COL1A1 (Collagen Type I Alpha 1) were subsequently determined using Real-Time PCR and Immunofluorescence Cytochemistry (IFC). MMP14 mRNA levels were increased across all three treatment groups (CoCl_2_, TGF-β, and the combined CoCl_2_/TGF-β group) at 1, 3, and 6 h compared to the control. The study noted a temporal pattern: the increase was most pronounced at 1 h under CoCl₂-induced chemical hypoxia, shifted to a higher increase at 3 h under TGF-β treatment, and peaked with a further increase at 6 h in the combined CoCl_2_/TGF-β group (Fig. 3B).

Examination of COL1A1 mRNA expression showed a decrease at 1, 3, and 6 h under CoCl₂-induced chemical hypoxia. Conversely, the known TGF-β-upregulated gene displayed a significant increase at all three time points following TGF-β application. Notably, the combined CoCl_2_/TGF-β treatment resulted in a decreased expression of COL1A1 (Fig. 3C).

### Analysis of MMP14 Gene Expression at the Protein Level

**Fig. 4:**
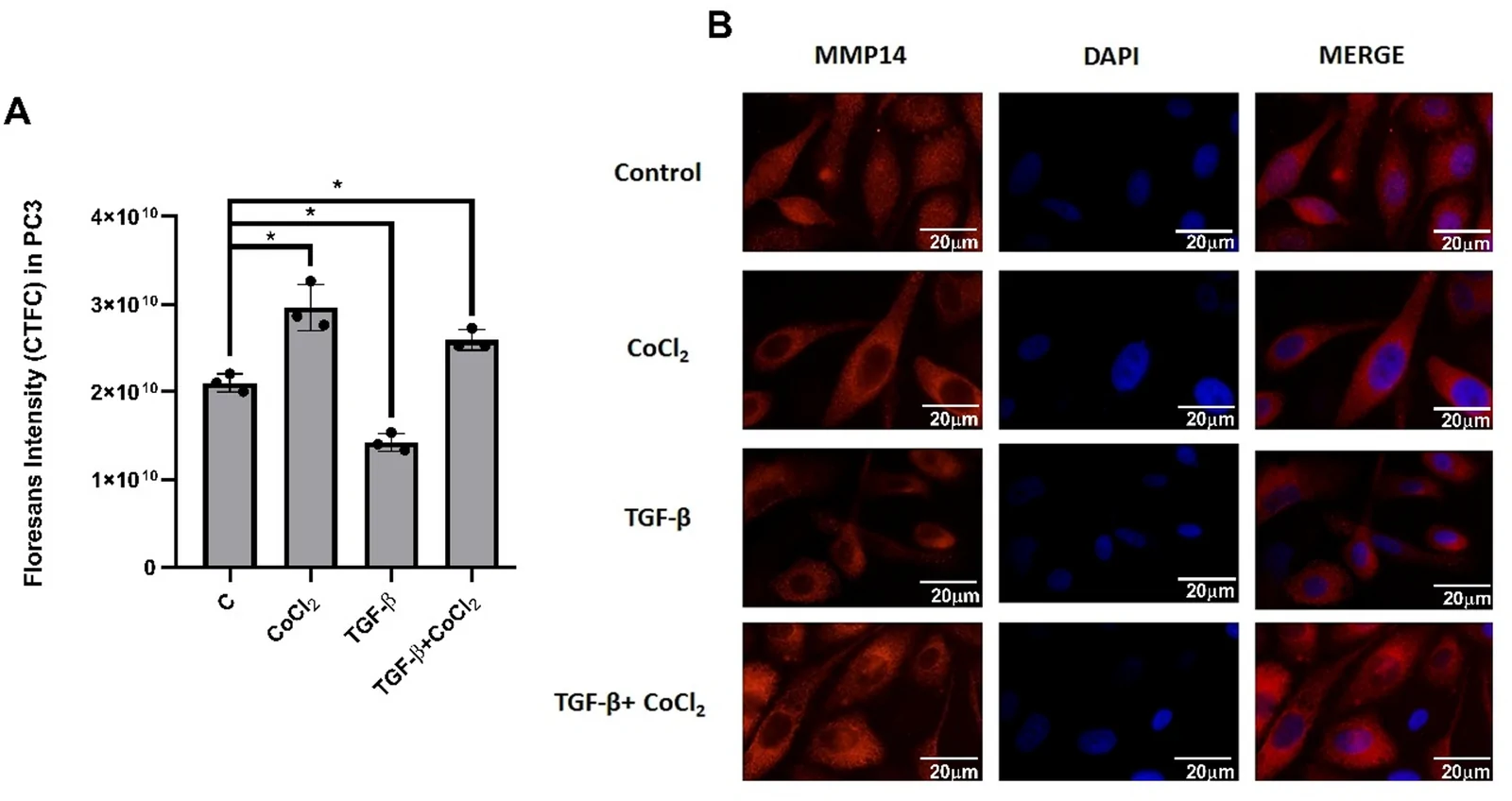
Analysis of MMP14 Protein Expression and Cellular Localization in PC3 Cells via Immunofluorescence Cytochemistry (IFC). Cells were exposed to Normoxia (control), Chemically Mimetic Hypoxia (CoCl_2_), TGF-β treatment, and the combined TGF-β/ CoCl₂ for 48 h. **A**: Quantitative Analysis of MMP14 Protein Expression. A graphical representation showing the relative protein expression level of MMP14. The signal intensity was quantified using image analysis software, comparing treated groups against the normoxic control. **B**: Representative Immunofluorescence Cytochemistry (IFC) Images. Images labeled with MMP14 represent cellular labeling of MMP14 protein. Images labeled with DAPI represent nuclear labeling of PC3 cells. Images labeled with MERGE represent superimposed MMP14 and DAPI images. (* *p* ≤ 0.05)

Immunofluorescence Cytochemistry (IFC) studies were conducted to analyze the MMP14 protein level following the mRNA expression analyses. Examination of IFC results at the 24-hour time point showed that MMP14 protein expression was not markedly altered in the TGF-β-treated group compared with the control group. In contrast, increased MMP14 protein expression was observed in both the CoCl₂-induced chemical hypoxia group and the combined CoCl₂/TGF-β treatment group. These findings suggest that CoCl₂-induced chemical hypoxia may contribute to increased MMP14 protein accumulation in PC3 cells at 24 h. Representative immunofluorescence images and quantitative analysis of MMP14 protein expression are presented in Fig. 4A–B.

## Discussion

The present study investigated the transcriptional regulation of MMP14 in PC3 prostate cancer cells under TGF-β stimulation and CoCl₂-induced chemical hypoxia. The results demonstrated that MMP14 expression was regulated in a context-dependent manner and that chemical hypoxia represented the dominant stimulus affecting MMP14 transcriptional activity, mRNA expression, and protein accumulation. Furthermore, promoter analyses indicated differential responses among deletion constructs, suggesting the contribution of distinct upstream regulatory regions to MMP14 responsiveness.

Functional promoter analyses demonstrated that all deletion constructs retained transcriptional activity, whereas the longest construct, P1 (− 1251/+75), exhibited the highest basal promoter activity. This observation suggests that upstream regulatory sequences located within the − 1251 to − 649 region may contribute to basal MMP14 transcriptional activity. Furthermore, treatment-dependent promoter responses revealed a region-specific regulation pattern. While P1 displayed increased activity under all experimental conditions, the shorter P2 and P3 fragments showed differential responses, particularly following TGF-β treatment. These findings suggest that the upstream promoter region may contain regulatory elements contributing to MMP14 responsiveness under TGF-β and hypoxia-associated signaling conditions.

The mRNA expression analyses further demonstrated that CoCl₂-induced chemical hypoxia markedly increased MMP14 expression in PC3 cells. Increased transcript levels were observed at early time points and were accompanied by elevated HIF-1α expression, supporting the establishment of chemically induced hypoxia-mimetic conditions. These findings are consistent with previous reports demonstrating that hypoxia and HIF-mediated signaling contribute to tumor invasion, extracellular matrix remodeling, and induction of metastasis-associated genes [11–13]. The sustained induction of MMP14 observed under CoCl₂ treatment, together with enhanced promoter activity, supports the involvement of hypoxia-responsive transcriptional mechanisms in MMP14 regulation.

In contrast to the pronounced effects of chemical hypoxia, TGF-β treatment alone did not induce a uniform response across all promoter constructs. However, combined CoCl₂/TGF-β treatment resulted in increased MMP14 transcriptional activity and mRNA expression, indicating that TGF-β may function as a context-dependent co-regulator under hypoxic stress conditions. Since the MMP14 promoter contains multiple putative SMAD binding regions, these observations suggest a possible interaction between TGF-β signaling and hypoxia-responsive pathways during MMP14 regulation.

Protein-level analyses performed by Immunofluorescence Cytochemistry (IFC) supported the transcriptional findings. While TGF-β treatment alone did not markedly alter MMP14 protein expression at 24 h, increased MMP14 accumulation was observed in both CoCl₂-treated and combined CoCl₂/TGF-β groups. The concordance between promoter activity, mRNA expression, and protein accumulation suggests that chemical hypoxia contributes substantially to MMP14 regulation in PC3 cells.

To evaluate the potential functional consequences of MMP14 induction, COL1A1 expression was also examined. TGF-β treatment alone increased COL1A1 expression, consistent with its established role in extracellular matrix deposition and remodeling [8, 24–28]. In contrast, reduced COL1A1 expression was observed in the CoCl₂-treated and combined CoCl₂/TGF-β groups, where MMP14 expression was elevated. This inverse expression pattern suggests a possible association between increased MMP14 activity and extracellular matrix remodeling under hypoxic conditions. However, additional functional studies are required to clarify the mechanistic relationship between MMP14 and COL1A1 regulation during tumor progression.

The synergistic induction of MMP14 observed in this study aligns with its role as a key driver of malignancy. Recent functional investigations have demonstrated that MMP14 overexpression directly promotes colony formation and accelerated cell growth, as seen in aggressive models like glioblastoma [29]. Our results characterize the regulatory axis (Hypoxia/TGF-β) that leads to this validated aggressive phenotype, providing a mechanistic basis for its transcriptional activation. While phenotypic changes such as increased motility remain to be directly validated through functional migration assays, our results establish the molecular foundation for such transitions.

In summary, the findings of this study indicate that MMP14 expression in PC3 cells is predominantly regulated by CoCl₂-induced chemical hypoxia, whereas TGF-β acts as a context-dependent co-regulator whose effects become more apparent under hypoxic conditions. Future studies involving inhibition experiments and Electrophoretic Mobility Shift Assays (EMSA) would be valuable to further characterize transcription factor binding events and clarify the molecular mechanisms governing MMP14 regulation.

## Supplementary Information

Below is the link to the electronic supplementary material.

Supplementary Material 1

## Data Availability

No datasets were generated or analysed during the current study.

## Abbreviations

ANOVA: Analysis of Variance
BSA: Bovine Serum Alumin
CoCl_2_: Cobalt Chloride
COL1A1: Collagen Type 1 Alpha 1
DAPI: 4’,6-Diamidino-2-phenyindole
ECM: Extracellular Matrix
EMT: Epithelial-Mesenchymal Transitin
EMSA: Electrophoretic Mobiliyt Shift Assay
FCS: Fetal Calf Serum
GC: Guanine and Cytosine
HIF-1α: Hypoxia-Inducible Factor-1 Alpha
HRE: Hypoxia Response Element
IFC: Immunofluoresence Cytochemistry
MMP: Matrix Metalloproteinase
MT1-MMP: Membrane-Type 1 Matrix Metalloproteinase
PBS: Phosphate-Buffered Saline
PCR: Polymerase Chain Reaction
qRT-PCR: Quantitative Real-Time Polymerase Chain Reaction
RNA: Rebinucleic Acid
SEAP: Secreted Alkaline Phosphatase
SMAD: Sma and Mad-related
T/A cloning: Thymine/Adenine cloning
TGF-β: Transforming Growth Factor-β
TSS: Transcription Start Site
MMP: Matrix Metalloproteinase
MMP14: Matrix Metalloproteinase-14
MT1-MMP: Mebrane-Type 1 Matrix Metalloproteinase
MTT: 3-(4,5-Dimethylthiazol-2-yl)-2,5-diphenyltetrazolium Bromide
