# Supplementary material for: The Dual Role of TGF-β and Hypoxia on MMP14-Mediated Invasion in PC3 Cells
Source: Cell Biochem Biophys. 2026 Jun 25;84(3):4025–34. doi: 10.1007/s12013-026-02102-3 (PMC13585980; doi:10.1007/s12013-026-02102-3)
Supplement: Supplementary file 1 [file 12013_2026_2102_MOESM1_ESM.docx]

**Supplementary Table 1.** Primer sequence list used in PCR

| Primer Name | Sequence | |
| --- | --- | --- |
| *Hβ-microglobulin F* | | 5’-TTTCTGGCCTGGAGGCTATC- 3’ |
| *Hβ-microglobulin R* | | 5’-CATGTCTCGATCCCACTTAACT--3’ |
| *MMP14 P1* | | 5’-AGATCTATATACCTCCCACAAACCTG-3’ |
| *MMP14 P2* | | 5’-AGATCTTTCCTCCCAGCGAGTTCCAATTAA |
| *MMP14 P3* | | 5’-AGATCTCCACAAAAAGGCAACTTAGAGGT |
| *MMP14 R1* | | 5’-GAGCTCTCAAAGTGCCTGTTTGCTCTTC-3’ |
| *MMP14 Exp F* | | 5’TTTTACGGCTTGCAAGTAACAGGCAAA-3’ |
| *MMP14 Exp R* | | 5’CATGGAAGCCCTCGGCAAAGAAGAT-3’ |
| *Col1A F* | | 5’-CTAGACATGTTCAGCTTTGTG-3’ |
| *Col1A R* | | 5’-GTTGTCGCAGACGCAGATCCG-3’ |
| *HIF-1α F* | | 5′-CCACCTATGACCT GCTTGGT-3’ |
| *HIF-1α R* | | 5′-TGTCCTGTGGTGACTTGTCC-3’ |
